# Complete genome sequence of *Gordonia rubripertincta* 112, a promising degrader of aromatic and aliphatic compounds

**DOI:** 10.1128/mra.00114-25

**Published:** 2025-05-28

**Authors:** Ekaterina Frantsuzova, Yulia Kocharovskaya, Anna Vetrova, Yanina Delegan, Alexander Bogun

**Affiliations:** 1 Institute of Biochemistry and Physiology of Microorganisms, Federal Research Center “Pushchino Scientific Center for Biological Research of Russian Academy of Sciences” (FRC PSCBR RAS) 133901 , Pushchino, Moscow Oblast, Russia; California State University San Marcos 14673, San Marcos, California, USA

**Keywords:** *Gordonia*, biodegradation, aromatic compounds, aliphatic compounds

## Abstract

The strain *Gordonia rubripertincta* 112 is a promising degrader of aromatic and aliphatic compounds. The genome of the strain was sequenced and completely assembled. It consists of a 5,108,631 bp circular chromosome (guanine-cytosine [GC] 67.34%) and two circular plasmids: pCP998 (99,888 bp, GC 64.81%) and pCP905 (90,599 bp, GC 65.89%).

## ANNOUNCEMENT

Members of the bacterial genus *Gordonia* are known for their ability to utilize pollutants with various chemical structures, e.g., alkanes (1), naphthalene (2), and thiophenes (3).

The strain *G. rubripertincta* 112 (IEGM 112) was isolated from soil in the 1980s. It can be found in the IEGM Regional Specialized Collection of Alkanotrophic Microorganisms (Perm, Russia) (the strain card G. rubripertincta IEGM 112) and in the collection of the Laboratory of the Physiology of Microorganisms, IBPM RAS (Pushchino, Moscow Region, Russia). The strain transforms benzoate, catechol, decane, and hexadecane (4). Sequencing of this strain was necessary for us to understand the genetic organization of the degradation mechanisms of these compounds.

For long-term storage, the strain was kept in glycerol (40%) at −70°С. For short-term maintenance, the strain was cultured on Lysogeny broth (LB) (5) agar plates at 27°C.

Genomic DNA was isolated from a fresh culture biomass (a colony) of *G. rubripertincta* 112 grown on LB agar using a DNeasy kit (Cat. No. 69506, Qiagen). The DNA concentration and quality were determined using a Qubit 4 Fluorometer and Nanodrop ND-1000 (Thermo Fisher Scientific). The long reads were generated with PromethION sequencing (Oxford Nanopore Technologies [ONT], UK). The sequencing libraries were prepared using the ligation sequencing kit SQK-LSK114-XL and native barcoding expansion kit SQK-NBD114.96 and run in R10.4.1 flow cell (FLO-MIN114, ONT). Reads were basecalled using Guppy v.6.5.7 using default parameters (high accuracy model, minimum quality value ≥7), which yielded a total of 1,101.9 Mb distributed in 172,617 reads (*N*50 is 12,581 bp).

The same DNA sample was sequenced on an MGI platform (DNBSEQ-G400) using the DNBSEQ-G400RS High-throughput Sequencing Set (FCL PE150) (2 × 150 bp). A paired-end library was prepared with the MGIEasy Universal DNA Library Prep Set. We obtained 11,794,002 paired-end reads (4). Quality control was performed using FastQC v.0.11.5 (6) and NanoPack v.1.1.0 (7). The MGI and Nanopore reads were used for hybrid assembly with SPAdes v.3.15.4 (8). The Nanopore reads were assembled into three contigs using Flye v.2.9.1 (9). Next, SPAdes contigs were combined into replicons using SnapGene v.6.0 with Flye data as reference. The MGI reads were used to correct Nanopore errors using Bowtie2 v.2.3.4.3 (very sensitive local mode) (10) and Pilon v.1.24 (11). The completeness and contamination of the assembly were determined using CheckM v.1.2.2 (12). Default parameters were used for all software unless otherwise specified.

The complete genome of *G. rubripertincta* strain 112 (completeness 98.30%, contamination 0.45%) consists of a 5,108,631 bp circular chromosome (guanine-cytosine [GC] 67.34%) and two circular plasmids: pCP998 (99,888 bp, GC 64.81%) and pCP905 (90,599 bp, GC 65.89%). Chromosome and plasmid circularization was specified by end overlapping and using Tablet v.1.21.02.08 (13).

To identify the strain to species, average nucleotide identity (ANI) (14) and digital DNA-DNA hybridization (dDDH) (15) parameters were used. ANI and dDDH values with the type strain of *G. rubripertincta* NBRC101908 (NZ_BAHB01000134.1) were 99.95% and 99.40%, respectively.

The strain 112 genome was annotated with the National Center for Biotechnology Information Prokaryotic Genome Annotation Pipeline v.4.6 (16). The genome contains 4,909 genes in total, 4 complete rRNA clusters, 49 tRNAs, and three ncRNAs. The strain bears a number of catabolic genes for aromatic compounds, alkanes, and steroid transformation.

## Data Availability

This genome project has been deposited at GenBank under BioSample SAMN34123077, BioProject PRJNA953757, GenBank accession number CP178555-CP178557, and SRA accession numbers SRX27560202 for MGI and SRX27560198 for Oxford Nanopore data.

## References

[1] Lo Piccolo L, De Pasquale C, Fodale R, Puglia AM, Quatrini P. 2011. Involvement of an alkane hydroxylase system of Gordonia sp. strain SoCg in degradation of solid n-alkanes. Appl Environ Microbiol 77:1204–1213. doi:10.1128/AEM.02180-10 21183636 PMC3067205

[2] Lin CL, Shen FT, Tan CC, Huang CC, Chen BY, Arun AB, Young CC. 2012. Characterization of Gordonia sp. strain CC-NAPH129-6 capable of naphthalene degradation. Microbiol Res 167:395–404. doi:10.1016/j.micres.2011.12.002 22240034

[3] Alves L, Marques S, Matos J, Tenreiro R, Gírio FM. 2008. Dibenzothiophene desulfurization by Gordonia alkanivorans strain 1B using recycled paper sludge hydrolyzate. Chemosphere 70:967–973. doi:10.1016/j.chemosphere.2007.08.016 17897697

[4] Frantsuzova E, Bogun A, Solomentsev V, Vetrova A, Streletskii R, Solyanikova I, Delegan Y. 2023. Whole genome analysis and assessment of the metabolic potential of Gordonia rubripertincta strain 112, a degrader of aromatic and aliphatic compounds. Biology (Basel) 12:721. doi:10.3390/biology12050721 37237534 PMC10215345

[5] BERTANI G. 1951. Studies on lysogenesis. I. The mode of phage liberation by lysogenic Escherichia coli. J Bacteriol 62:293–300. doi:10.1128/jb.62.3.293-300.1951 14888646 PMC386127

[6] Andrews S. 2010. FastQC: a quality control tool for high throughput sequence data. Available from: http://www.bioinformatics.babraham.ac.uk/projects/fastqc

[7] De Coster W, D’Hert S, Schultz DT, Cruts M, Van Broeckhoven C. 2018. NanoPack: visualizing and processing long-read sequencing data. Bioinformatics 34:2666–2669. doi:10.1093/bioinformatics/bty149 29547981 PMC6061794

[8] Bankevich A, Nurk S, Antipov D, Gurevich AA, Dvorkin M, Kulikov AS, Lesin VM, Nikolenko SI, Pham S, Prjibelski AD, Pyshkin AV, Sirotkin AV, Vyahhi N, Tesler G, Alekseyev MA, Pevzner PA. 2012. SPAdes: a new genome assembly algorithm and its applications to single-cell sequencing. J Comput Biol 19:455–477. doi:10.1089/cmb.2012.0021 22506599 PMC3342519

[9] Kolmogorov M, Yuan J, Lin Y, Pevzner PA. 2019. Assembly of long, error-prone reads using repeat graphs. Nat Biotechnol 37:540–546. doi:10.1038/s41587-019-0072-8 30936562

[10] Langmead B, Salzberg SL. 2012. Fast gapped-read alignment with bowtie 2. Nat Methods 9:357–359. doi:10.1038/nmeth.1923 22388286 PMC3322381

[11] Walker BJ, Abeel T, Shea T, Priest M, Abouelliel A, Sakthikumar S, Cuomo CA, Zeng Q, Wortman J, Young SK, Earl AM. 2014. Pilon: an integrated tool for comprehensive microbial variant detection and genome assembly improvement. PLoS One 9:e112963. doi:10.1371/journal.pone.0112963 25409509 PMC4237348

[12] Parks DH, Imelfort M, Skennerton CT, Hugenholtz P, Tyson GW. 2015. CheckM: assessing the quality of microbial genomes recovered from isolates, single cells, and metagenomes. Genome Res 25:1043–1055. doi:10.1101/gr.186072.114 25977477 PMC4484387

[13] Milne I, Stephen G, Bayer M, Cock PJA, Pritchard L, Cardle L, Shaw PD, Marshall D. 2013. Using Tablet for visual exploration of second-generation sequencing data. Brief Bioinformatics 14:193–202. doi:10.1093/bib/bbs012 22445902

[14] Yoon SH, Ha SM, Lim JM, Kwon SJ, Chun J. 2017. A large-scale evaluation of algorithms to calculate average nucleotide identity. Antonie Van Leeuwenhoek 110:1281–1286. doi:10.1007/s10482-017-0844-4 28204908

[15] Meier-Kolthoff JP, Auch AF, Klenk HP, Göker M. 2013. Genome sequence-based species delimitation with confidence intervals and improved distance functions. BMC Bioinformatics 14:60. doi:10.1186/1471-2105-14-60 23432962 PMC3665452

[16] Tatusova T, DiCuccio M, Badretdin A, Chetvernin V, Nawrocki EP, Zaslavsky L, Lomsadze A, Pruitt KD, Borodovsky M, Ostell J. 2016. NCBI prokaryotic genome annotation pipeline. Nucleic Acids Res 44:6614–6624. doi:10.1093/nar/gkw569 27342282 PMC5001611

